# Supplementary material for: Identification of photoperiod-induced specific miRNAs in the adrenal glands of Sunite sheep (Ovis aries)
Source: Front Vet Sci. 2022 Jul 22;9:888207. doi: 10.3389/fvets.2022.888207 (PMC9354845; doi:10.3389/fvets.2022.888207)
Supplement: Supplementary file 1 [file Data_Sheet_1.ZIP › Supplementary file/miRDeep2 file.zip!/miRDeep2 file/Novel_Structure/SPLP42A3/Novel_257.pdf]

Provisional ID	: NC_019482.2_36846
Score total	: 1.7
Score for star read(s)	: -1.3
Score for read counts	: 0
Score for mfe	: 1.4
Score for randfold	: 1.6
Score for cons. seed	:
Total read count	: 399
Mature read count	: 399
Loop read count	: 0
Star read count	: 0

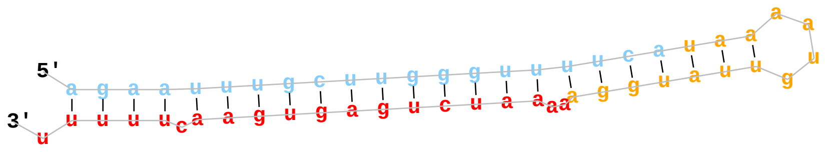

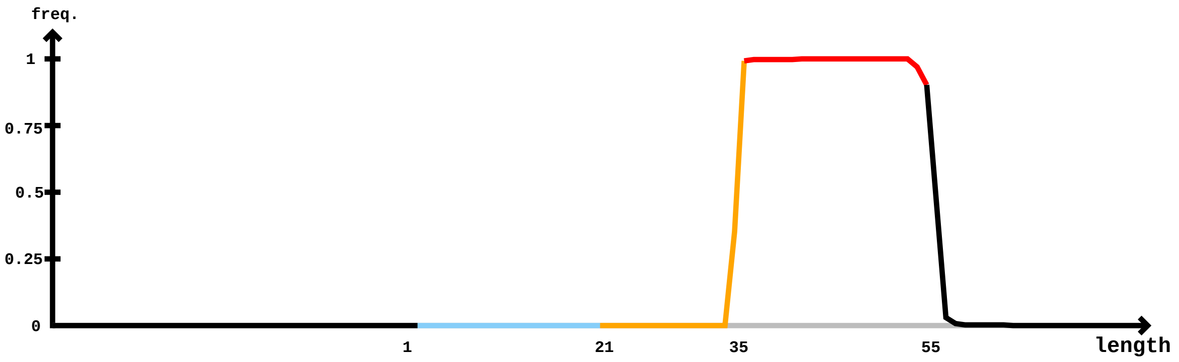

Star Mature

```
5'- aaccauuauauacagaugauuuagugaguuggccaaagaauuugcuuggguuuucauaaaauguuauggaaaaaucugagugaacuuuuuggccaacggaauauuuuucucc   -3'    exp
    ...(((((.............))))).((((((((((((.(((((((((((((((((((....)))))))..))))))))))))).))))))))))))((((.....))))..     reads     mm      sample
    .....................................................................aaaaaucugaAugaacuuu........................     12        1       seq
    .....................................................................aaaaaucugagugaacuuuu.......................     1         0       seq
    .....................................................................aaaaaucugaAugaacuuuu.......................     26        1       seq
    .....................................................................aaaaaucugaAugaacuuuuu......................     78        1       seq
    .....................................................................aaaaaucugaAugaacuuuuug.....................     24        1       seq
    ......................................................................aaaaucugaAugaacuuuuu......................     247       1       seq
    ......................................................................aaaaucugagugaacuuuuugg....................     9         0       seq
    .......................................................................aaaucugagugaacuuuuuggc...................     2         0       seq
    ............................................................................ugagugaacuuuuuggccaacU..............     1         1       seq
```
